# From ALL to Myeloid and NK Malignancies: Operationalizing “ASNS-Low” for L-Asparaginase Repurposing and Combination Therapy

**DOI:** 10.3390/biom16060792

**Published:** 2026-05-27

**Authors:** Toshiyuki Kitoh

**Affiliations:** Laboratory of Pediatrics, School of Pharmacy, Aichi Gakuin University, Nagoya 464-8650, Japan; tkitoh@dpc.agu.ac.jp

**Keywords:** L-asparaginase, asparagine synthetase, ASNS-low, acute myeloid leukemia, NK/T-cell lymphoma, biomarker, intracellular flow cytometry, proteomics, companion diagnostic, combination therapy

## Abstract

L-asparaginase (ASNase) is a paradigmatic amino-acid depletion therapy that induces systemic asparagine starvation and remains foundational in acute lymphoblastic leukemia (ALL). Amino-acid metabolism constitutes a fundamental therapeutic vulnerability in hematologic malignancies, yet the determinants of response to systemic asparagine depletion remain incompletely defined. Asparagine synthetase (ASNS) regulates intracellular asparagine biosynthesis and functions as a stress-responsive metabolic node embedded within adaptive nutrient-sensing pathways. Emerging transcriptomic and proteomic evidence demonstrates that reduced ASNS expression is enriched in biologically distinct subsets of acute myeloid leukemia (AML), particularly those characterized by immature differentiation states and cytogenetic features associated with metabolic fragility, including inv(16) and chromosome 7-associated disease. Clinical experience in natural killer/T-cell (NK/T-cell) neoplasms provides proof-of-principle that enzymatic asparagine depletion can achieve durable therapeutic efficacy in tumors intrinsically dependent on extracellular amino-acid supply, establishing extranodal NK/T-cell lymphoma (ENKTL) as a mechanistically aligned anchor indication beyond acute lymphoblastic leukemia. Integrative molecular analyses further indicate that ASNS deficiency functions as a permissive rather than deterministic biomarker, with therapeutic response modulated by lineage-specific metabolic wiring, adaptive stress signaling, and microenvironmental nutrient buffering. Advances in protein-anchored diagnostic platforms, including intracellular flow cytometry and quantitative proteomics, now enable operationalization of ASNS as a clinically actionable stratification marker. Mechanistic studies also suggest that amino-acid depletion may interact with apoptotic signaling networks, supporting rational combination strategies with targeted agents such as BCL-2 inhibitors. Collectively, these findings support a conceptual framework in which ASNS-low defines a context-dependent metabolic vulnerability rather than a uniform disease-wide predictor, underscoring the need for prospective biomarker-enriched clinical trials to establish ASNS-guided amino-acid depletion as a precision oncology strategy across heterogeneous myeloid and lymphoid malignancies.

## 1. Introduction and Scope

**Figure 1:**
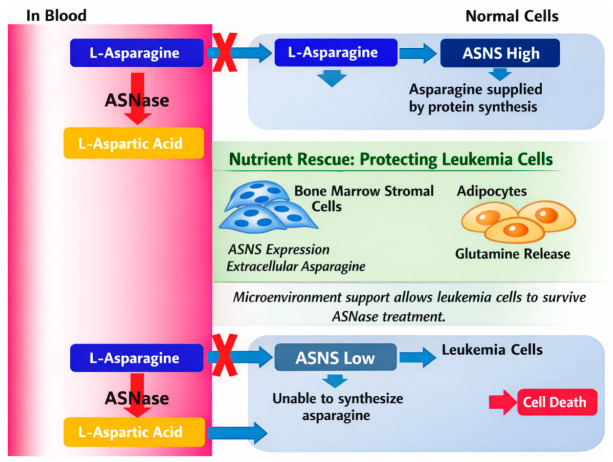
Context-dependent metabolic vulnerability of ASNS-low leukemia during ASNase therapy. ASNS-low leukemic cells are intrinsically susceptible to systemic asparagine depletion, but this effect may be attenuated in vivo by microenvironmental nutrient rescue. Bone marrow stromal cells can replenish extracellular asparagine, and adipocytes can support leukemic survival through glutamine release. In parallel, amino-acid depletion may suppress cap-dependent translation, reduce MCL-1 expression, and increase susceptibility to BCL-2 inhibition. The schematic therefore depicts ASNS-low as a prerequisite-like, but non-sufficient metabolic vulnerability shaped by both intrinsic and extrinsic determinants.

L-asparaginase (ASNase) is a paradigmatic amino-acid depletion therapy that exploits tumor dependence on extracellular asparagine when compensatory de novo synthesis is limited. Extending ASNase beyond canonical acute lymphoblastic leukemia (ALL) requires biomarker logic that is both clinically honest and operationally testable. This review frames “ASNS-low” (low or absent asparagine synthetase, ASNS) as a prerequisite-like state for ASNase benefit (Q requires P), while explicitly acknowledging that ASNS-low alone rarely guarantees response because lineage programs, treatment-induced metabolic rewiring, microenvironmental rescue, and exposure/toxicity constraints modulate realized benefit (Figure 1).

## 2. A Biomarker Logic That Is Safe to Claim: Necessary-like vs. Sufficient

Across experimental systems, ASNS protein content correlates inversely with ASNase sensitivity more consistently than ASNS mRNA, and perturbation experiments support causality (ASNS overexpression induces resistance; knockdown increases sensitivity) [1]. In contrast, multiple studies show that ASNS mRNA levels alone can fail to predict ASNase response in pediatric ALL contexts, arguing against a portable transcript-only cutoff [2,3,4]. Context dependence is not a footnote: in TEL-AML1-negative pediatric ALL, higher ASNS expression was linked to resistance, whereas this relationship did not generalize to TEL-AML1positive cases [3].

For review writing and for future companion diagnostic (CDx) work, the clinically defensible claim is therefore asymmetric: ASNS-low is best treated as a necessary-like vulnerability state (a prerequisite for meaningful benefit in selected contexts), whereas sufficiency must be earned by first applying explicit minimal context filters and then companion biomarkers. Mechanistically, sufficiency fails when (i) ASNS is inducible under amino-acid stress, (ii) effective drug exposure is not achieved or maintained, or (iii) extrinsic asparagine supply rescues tumor cells despite tumor-intrinsic ASNS-low.

## 3. What Counts as “Efficacy” for ASNase Repurposing

Operationalization requires endpoint discipline that aligns in vitro response metrics with clinically meaningful outcomes. In vitro and ex vivo systems typically quantify ASNase response using viability and apoptosis readouts together with summary metrics such as IC_50_/EC_50_, LD_70_, and AUC/DSS. In childhood AML, large ex vivo pharmacologic datasets demonstrated marked FAB-dependent heterogeneity in ASNase sensitivity. Median LD_70_asp values (U/mL) differed substantially across FAB subtypes, with relatively low values observed in M0 (0.76), M1 (0.46), M4 (1.18), and M5 (1.35), whereas M2, M3, and M7 samples exhibited markedly higher LD_70_asp values approaching the upper assay limit. Notably, the distribution of LD_70_asp among M1 cases was comparable to that observed in childhood ALL, indicating a biologically meaningful degree of asparagine dependence in this subset. Collectively, these findings support the concept that selected AML subtypes, including M1, M4, and M5, may retain intrinsic sensitivity to ASNase rather than representing uniformly resistant disease entities [5].

Clinically, therapeutic “efficacy” of L-asparaginase (ASNase) should be defined not solely by early cytoreduction but by the integrated dimensions of response depth (objective response or remission) and durability (duration of response or event-free survival). A biologically coherent translational framework emerges when ASNS-low status is concordant with experimental susceptibility and sustained clinical benefit, as demonstrated most clearly in lymphoid malignancies. In acute myeloid leukemia (AML), however, the evidence supporting such alignment remains context-restricted and mechanistically heterogeneous.

Cytogenetic abnormalities involving chromosome 7 have been associated with reduced ASNS gene dosage and diminished protein expression, features that correlate with increased sensitivity to ASNase in preclinical models [6,7]. Functional perturbation studies further support a causal relationship between ASNS suppression and metabolic vulnerability to asparagine depletion, reinforcing the concept that intracellular asparagine biosynthetic capacity constrains therapeutic response [6]. Nevertheless, tumor-intrinsic susceptibility does not uniformly translate into durable clinical benefit.

A major confounder is the bone marrow microenvironment, which can attenuate ASNase efficacy through nutrient-rescue mechanisms. Mesenchymal stromal cells can upregulate ASNS and supply extracellular asparagine, thereby protecting leukemic blasts from enzymatic depletion [8,9]. Similarly, adipocyte-mediated glutamine release can buffer metabolic stress and sustain leukemic proliferation under ASNase exposure [10]. These findings underscore that therapeutic durability depends on systemic pharmacologic exposure and microenvironmental nutrient exchange rather than tumor-intrinsic ASNS status alone.

Experimental AML models further demonstrate that ASNase exerts antiproliferative and pro-apoptotic effects, with activation of apoptosis pathways correlating with low baseline ASNS expression and impaired adaptive metabolic responses [11,12]. Collectively, these data support a conceptual model in which ASNase activity in AML is governed by a convergence of genetic context, metabolic state, and microenvironmental modulation. Accordingly, ASNS-low should be interpreted as a prerequisite-like determinant of susceptibility rather than a sufficient predictor of durable response, emphasizing the need for biomarker-anchored clinical validation strategies.

## 4. Biological Rationale and Failure Modes: Why ASNS-Low Is Rarely Sufficient

ASNS is a stress-responsive metabolic enzyme embedded within the integrated stress response (ISR) and broader nutrient-sensing transcriptional programs. Under amino-acid deprivation, coordinated promoter occupancy by ATF4, ATF3, and C/EBPβ induces ASNS transcription, while unfolded protein response signaling can further reinforce ASNS upregulation. These adaptive mechanisms provide a biologically plausible escape pathway during systemic asparagine depletion and help explain why baseline ASNS deficiency does not uniformly translate into sustained therapeutic sensitivity [13,14].

Even when leukemic cells are intrinsically ASNS-low, extrinsic nutrient exchange can uncouple tumor ASNS status from effective asparagine depletion in vivo. Bone marrow mesenchymal stromal cells can express high levels of ASNS and locally replenish asparagine concentrations, thereby protecting leukemic blasts from ASNase-mediated cytotoxicity in co-culture models [9]. Adipocytes similarly promote leukemia cell survival through glutamine release, illustrating a microenvironmental nutrient-rescue mechanism that can function as a sufficiency breaker despite tumor-intrinsic metabolic vulnerability [10].

Consistent with this context-dependent framework, recent translational studies indicate that modulation of amino-acid availability can influence apoptotic signaling pathways central to therapeutic response. Amino-acid depletion, including ASNase-induced metabolic stress, can disrupt cap-dependent translation and reduce MCL-1 protein abundance, thereby enhancing susceptibility to BCL-2 inhibition. These findings support the concept that metabolic targeting strategies may operate not only as direct cytotoxic interventions but also as sensitizing mechanisms that reshape apoptotic dependency networks [15] (Figure 1).

However, the clinical utility of ASNS as a biomarker must be interpreted within a context-dependent framework integrating adaptive stress competence, lineage-specific metabolic programs, microenvironmental nutrient rescue, and pharmacologic exposure heterogeneity. Inducible ASNS re-expression via ATF4 signaling, glutamine buffering capacity, and variability in achieved ASNase exposure can each limit durability even when baseline ASNS expression is low.

Formulation enzymology introduces an additional layer of complexity. ASNase preparations retaining glutaminase co-activity can contribute to more durable preclinical antileukemic effects, implying that the effective pharmacologic mechanism may extend beyond isolated asparagine depletion and may interact with glutamine-axis metabolic context [16]. Finally, asparagine itself functions as an amino-acid exchange factor capable of regulating global amino-acid homeostasis and proliferative signaling, suggesting that asparagine depletion exerts network-level metabolic effects beyond a single biosynthetic pathway [17].

## 5. How to Prove “ASNS-Low”: Assay Modalities and What They Really Measure

This review organizes “ASNS-low” operationalization by assay modality, emphasizing analytical validity (what is measured), clinical validity (association with response), and deployability.

### 5.1. Enzymatic Activity Assays (Reference Standard)

Historically, ASNS-low was defined functionally by measuring ATP-dependent asparagine synthesis from aspartate with glutamine as nitrogen donor. Modern methods include radiotracer product assays, chromatography-based product quantification, and coupled AMP-production formats; activity assays are closest to causality but are constrained by specimen requirements, throughput, and cross-laboratory standardization, and thus are best positioned as a reference assay for calibrating scalable biomarkers [18,19,20].

### 5.2. Immunostaining (ICC/IHC; FFPE-First Feasibility)

Immunostaining enables assessment of ASNS protein abundance within its histologic and cellular context and is therefore inherently compatible with companion diagnostic (CDx) development workflows. However, reliable clinical deployment requires rigorous antibody validation, strict pre-analytic control (including fixation and processing conditions), and standardized scoring frameworks to ensure reproducibility across laboratories.

Protein-level designation of “ASNS-negative/low” by immunostaining provides clinically anchored, albeit largely case-based, support for apparent clinical sensitivity to ASNase in selected AML contexts [7,21,22]. Early translational clinical observations demonstrated that refractory AML cases harboring chromosome 7 abnormalities and lacking detectable ASNS protein by immunohistochemistry exhibited objective responses to ASNase-containing salvage regimens [7]. Similarly, pediatric AML and myeloid sarcoma cases with ASNS-negative immunostaining have shown early clinical improvement following ASNase exposure, reinforcing the biological plausibility of ASNS deficiency as a functional vulnerability marker [21].

Additional ICC/IHC-based observations across the AML disease spectrum support this paradigm, indicating that protein-level ASNS assessment may provide actionable biological stratification even when transcriptomic data are inconclusive [23,24,25,26,27]. Notably, early multi-institutional clinical experience demonstrated that leukemia cells from multiple FAB M0/M1/M5 cases frequently lacked detectable ASNS staining and that ASNase-containing regimens could induce objective responses in refractory acute non-lymphocytic leukemia, further supporting the predictive potential of immunostaining-based ASNS assessment [22].

These clinical observations are conceptually consistent with large ex vivo pharmacologic datasets in childhood AML demonstrating marked FAB-dependent heterogeneity in ASNase sensitivity, in which AML M1, M4, and M5 samples showed relatively greater susceptibility compared with other subtypes [5]. Together, these convergent clinical and pharmacologic data support a model in which protein-level ASNS deficiency identifies a biologically enriched subset of AML characterized by context-dependent but potentially therapeutically exploitable sensitivity to systemic asparagine depletion.

Practical implementation therefore depends on engineering diagnostic workflows that minimize ambiguity near background staining thresholds and anchor classification cutoffs to clinical outcomes or orthogonal functional correlates, including pharmacologic response metrics.

### 5.3. Intracellular Flow Cytometry (Fresh-First, Single-Cell Quantification)

Intracellular ASNS flow provides a clinically practical shift from transcript surrogates to a direct, single-cell protein phenotype that can be integrated into routine leukemia gating on PB/BM specimens. A key enabling advance was the development of flow-compatible anti-ASNS monoclonal antibodies, among which Z5808 MoAb (IgG2a isotype) emerged as the lead reagent for quantitative intracellular testing. In the Hybridoma study, Z5808 and Z5801 both showed specific intracellular staining shifts, but Z5808 exhibited higher reactivity than Z5801 on flow cytometric analysis and recognized endogenous ASNS in K562 cells, supporting analytical specificity and sensitivity for clinical translation [27]. The same platform further enabled QIFIKIT-based calibration of flow signal to antibody binding capacity (ABC), with an estimated dynamic range of approximately 5800 ASNS molecules/cell in K562 versus 40 molecules/cell in MOLT-4, demonstrating that ASNS can be quantified across biologically relevant high- and low-expression states [27]. In subsequent leukemia-focused application studies, cytosolic flow using Z5808 quantified ASNS as ΔMFI and MFI ratio against an isotype control, and these protein-level metrics were inversely correlated with L-asparaginase sensitivity in cell lines and primary AML samples; cell line-anchored candidate cutoffs (<25 for ΔMFI and <1.8 for MFI ratio) were also proposed for enrichment use [28]. Clinical deployability depends on controlling dynamic range near background, explicit background correction (e.g., ΔMFI or MFI ratio), and adherence to standardization/reporting frameworks (MIFlowCyt; EuroFlow-like instrument harmonization) for cross-run and cross-site comparability [26,27,28,29,30].

### 5.4. Proteomics (Reverse-Phase Protein Array (RPPA) and Mass Spectrometry (MS)-Based Proteomic Profiling)

Proteomics can quantify ASNS at scale, but large-cohort proteome data also show why proteomic context can matter as much as the ASNS axis itself. In 810 newly diagnosed AML cases profiled by RPPA, heterogeneous ASNS abundance was observed, with inv(16) cases among the lowest, and ASNS showing prognostic and therapy-interaction signals [31]. Mass spectrometry complements antibody proteomics by enabling precise quantification; targeted Liquid chromatography–tandem mass spectrometry (LC–MS/MS) has quantified ASNS in circulating leukemia cells [32], AQUA-style strategies support absolute quantification [33], and multicenter harmonization frameworks for distributed proteotype analysis provide a translational path for robust measurement [34]. Dissemination of validated assays via resources such as the CPTAC Assay Portal can reduce interlaboratory variability and accelerate fit-for-purpose deployment [35].

### 5.5. ASNS mRNA and Promoter Methylation (State Markers; Context-Dependent)

ASNS mRNA alone is frequently discordant with protein and can fail as a universal predictor [1,2,3,4]. Nonetheless, mRNA can be clinically informative in defined contexts: in extranodal natural killer/T-cell lymphoma, nasal type (ENKTL) models, ASNS expression correlated with ASNase IC_50_ and functional manipulation of ASNS altered response in vitro and in vivo [36]. Epigenetic silencing provides a stronger “state” marker in selected lineages; ASNS promoter methylation correlated with ASNase sensitivity in T-ALL cell lines and patient-derived xenografts [37]. Reviews emphasize that epigenetic and post-transcriptional regulation can decouple mRNA from protein abundance, supporting a protein-anchored and state-aware operational definition of ASNS-low [38].

### 5.6. Lineage- and Cytogenetics-Dependent ASNS Expression in AML: Integrative Transcriptomic and Proteomic Evidence

**Figure 2:**
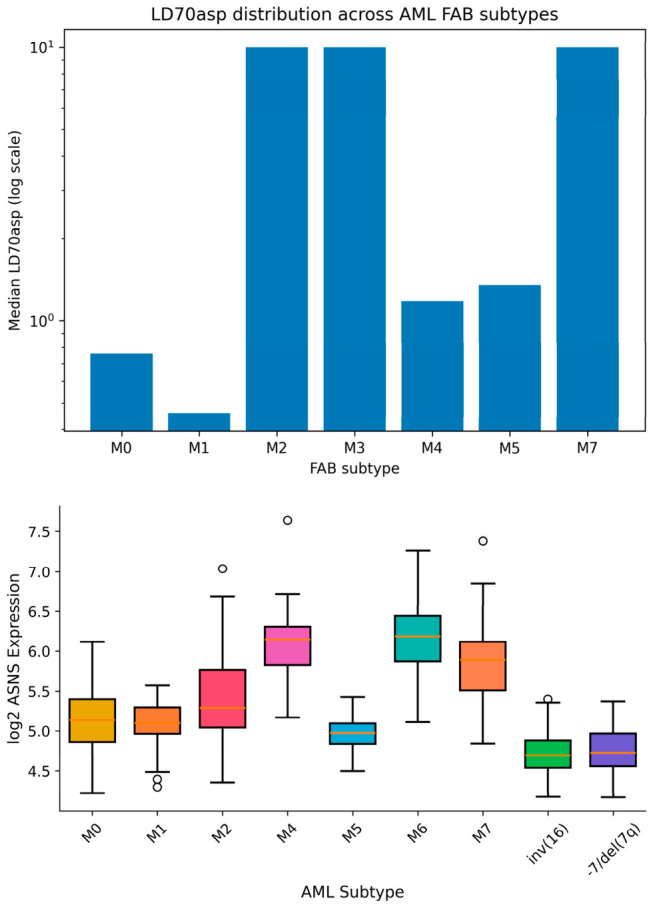
Integrated representation of subtype-dependent L-asparaginase sensitivity and ASNS expression in AML. The **upper panel** shows FAB-specific LD_70_asp distributions, indicating pharmacologic heterogeneity in ASNase sensitivity. The **lower panel** shows corresponding ASNS expression patterns across AML subtypes and selected cytogenetic categories, supporting the concept that reduced ASNS expression contributes to metabolically defined ASNase-sensitive AML subsets. Boxplots display median, interquartile range, and 1.5× interquartile range. Abbreviations: AML, acute myeloid leukemia; ASNS, asparagine synthetase; FAB, French–American–British classification.

Integrative analysis of publicly available AML transcriptomic cohorts, including datasets deposited in the Gene Expression Omnibus (GEO), demonstrates substantial heterogeneity in ASNS expression across FAB-defined and cytogenetically stratified AML subsets. Immature and monocytic AML subtypes (FAB M0, M1, and M5) consistently exhibit relatively low ASNS expression, suggesting reduced intrinsic asparagine biosynthetic capacity and increased reliance on extracellular amino-acid supply [30]. In contrast, more differentiated AML subtypes, including myelomonocytic (M4) and erythroid-lineage AML (M6), display comparatively higher ASNS expression, indicative of increased metabolic autonomy. Megakaryoblastic AML (M7) shows intermediate expression levels, consistent with lineage-dependent metabolic heterogeneity (Figure 2).

This transcriptional variability parallels pharmacologic observations demonstrating marked FAB-dependent heterogeneity in L-asparaginase sensitivity, thereby supporting a biologically coherent model linking metabolic phenotype to therapeutic response (Figure 2) [29]. Notably, cytogenetic subgroups associated with metabolic vulnerability, including core-binding factor AML with inv(16) and chromosome 7-associated abnormalities (−7/del(7q)), exhibit significantly reduced ASNS expression relative to other AML categories [31]. Collectively, these findings indicate that ASNS-low represents a lineage- and cytogenetics-contextual metabolic vulnerability rather than a uniform predictive biomarker across AML (Table 1).

This lineage-dependent heterogeneity is further supported by large-scale proteomic profiling studies. Reverse-phase protein array analyses encompassing large cohorts of newly diagnosed AML cases have demonstrated substantial inter-patient variability in ASNS protein abundance and have identified associations between elevated ASNS expression and inferior overall survival [32]. These findings suggest that enhanced adaptive metabolic stress buffering may contribute to disease aggressiveness and therapeutic resistance.

## 6. Head-to-Head Method Comparison and an Indication-Specific CDx Pathway

“ASNS-low” is not a platonic truth; its performance (sensitivity/specificity) must be estimated against a declared reference endpoint (protein, functional drug sensitivity, or clinical response). For binary outputs, performance evaluation should follow established qualitative-test frameworks, including imprecision characterization (C5/C95), sensitivity/specificity estimation, stability, and interference testing [39].

For immunohistochemical assays, analytic validation and revalidation should follow CAP guidance, with harmonized controls and scoring systems to reduce interobserver variability [40,41]. Regulatory framing for CDx emphasizes tests that provide information essential for the safe and effective use of a therapeutic product and expects analytical and clinical validation commensurate with the intended labeling claim [42,43,44]. In Europe, CDx development must be planned under IVDR and related consultation procedures involving EMA [45,46], with laboratory quality systems aligned to ISO 15189 [47].

**Figure 3:**
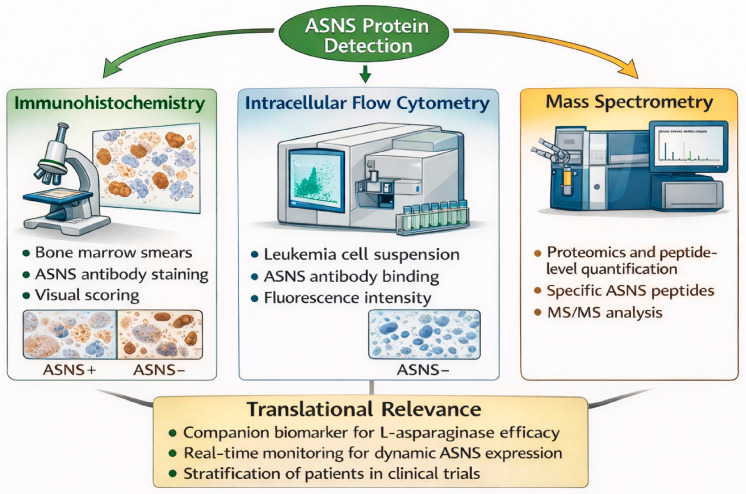
Diagnostic framework for ASNS as a companion biomarker. Schematic overview of protein-anchored ASNS assessment modalities: immunohistochemistry (IHC) for FFPE/bone marrow smear-based scoring, intracellular flow cytometry for rapid single-cell quantification in leukemia suspensions, and mass spectrometry for peptide-level proteomic quantification. Together, these platforms support companion biomarker use for L-asparaginase-based therapy, enable monitoring of dynamic ASNS expression, and facilitate patient stratification in biomarker-enriched clinical trials. Abbreviations: ASNS, asparagine synthetase; IHC, immunohistochemistry; MS/MS, tandem mass spectrometry.

A pragmatic near-term pathway is “protein-first screen + reflex,” tailored to indication: (i) ENKTL: FFPE-first ASNS IHC as the primary platform with prespecified binary + semi-quantitative scoring and CAP-grade validation, reflexing to RNA/methylation for equivocal cases or multi-site harmonization; (ii) AML: fresh-first intracellular ASNS flow on blasts (ΔMFI) where feasible, with reflex to targeted qPCR calibrated to flow when necessary, and initial use restricted to trial enrollment or protocolized salvage until prospective durability data accrue (Table 2). Diagnostic framework for ASNS as a companion biomarker is shown in Figure 3.

## 7. Indication Map: Where ASNS-Low-Guided ASNase Development Is Most Defensible

A review that “operationalizes” ASNS-low should rank indications by translational readiness: accumulation of (i) mechanistically coherent experimental data and (ii) clinically accumulated responsiveness with durability.

### 7.1. ENKTL as the Anchor Indication

ENKTL provides the strongest non-ALL alignment between mechanism and established clinical activity. ASNase-containing regimens have demonstrated major activity in advanced, relapsed, or refractory ENKTL, including the phase II SMILE study [48] and the AspaMetDex regimen [49]. Pegaspargase (A recombinant, pegylated form of E. coli–derived L-asparaginase)-containing regimens such as P-GemOx have also shown activity in ENKTL [50]. Within this disease, ASNS biology is plausibly a resistance/benefit modifier rather than a simplistic gate: ASNS/AsnS expression is associated with ASNase sensitivity in ENKTL models, and manipulation of ASNS can alter response [36]. Additional preclinical work demonstrates selective apoptosis of NK-cell tumors by ASNase, supporting mechanistic coherence for NK-lineage targeting [51], and case-based clinical reports illustrate activity of ASNase formulations in NK/T lymphoma contexts [52,53].

### 7.2. AML as a High-Value but Context-Restricted Extension

AML is the highest-impact frontier for ASNase repurposing but must be framed conservatively because metabolic heterogeneity and niche dependence can dilute tumor-intrinsic signals. A mechanistically coherent subset exists: chromosome 7 lesions and reduced ASNS gene/protein expression are linked to increased ASNase cytotoxicity, and ASNS knockdown increases sensitivity [6]. Clinical observations consistent with “apparent clinical sensitivity” have been reported in refractory AML with chromosome 7 abnormalities treated with ASNase salvage [7] and in selected pediatric AML-spectrum cases with ASNS-negative immunostaining and early responses [21,22,23,24,25]. However, the marrow niche can attenuate ASNase effects and is a decisive confounder for durability [8,9,10]. Accordingly, AML development should be combination-forward and prospective, with separate reporting of CR vs. CRi and emphasis on durability endpoints.

One clinically relevant example of combination logic is venetoclax plus pegcrisantaspase, which showed synergistic antileukemic activity in complex-karyotype AML models and clinical-correlative work [15]. Reviews of glutamine metabolism in AML further motivate amino-acid depletion strategies and highlight opportunities and challenges for systemic glutamine/asparagine targeting [54].

Notably, emerging clinical observations indicate that venetoclax-based therapeutic strategies may confer differential survival benefit according to ASNS expression status, underscoring the complex and context-dependent relationship between amino-acid metabolism and apoptotic vulnerability in AML. Taken together, integrated transcriptomic, proteomic, and translational clinical evidence supports a model in which ASNS expression reflects differentiation-state-dependent metabolic wiring that shapes both intrinsic susceptibility to amino-acid depletion and adaptive responses to targeted therapy (Table 3).

This table summarizes transcriptomic, proteomic, and translational evidence showing that ASNS expression varies across AML subsets and may define context-dependent metabolic vulnerability. Lower ASNS expression is enriched in immature/monocytic FAB subtypes (M0, M1, and M5), as well as inv(16) and −7/del(7q) AML, whereas M4 and M6 more often show higher ASNS expression. Together, these data support interpretation of ASNS-low as a lineage- and cytogenetics-contextual vulnerability rather than a universal predictor of response.

### 7.3. Beyond ENKTL and AML: Hypothesis-Generating Extensions

Myeloid/NK cell precursor acute leukemia (MNKPL) represents a rare but mechanistically aligned entity in which ASNase-based strategies have produced clinically meaningful responses in case-based reports, including remission enabling transplantation after failure of conventional approaches [25] and AML-oriented chemotherapy regimens incorporating ASNase [23]. Although the evidence base remains limited by rarity and heterogeneous treatment backbones, MNKPL provides a high-yield setting for biomarker-anchored prospective collection (protein-level ASNS, inducibility, and epigenetic state) to test whether profound ASNS deficiency translates into durable vulnerability under controlled exposure. Malignant lymphomas outside NK-lineage are heterogeneous with limited accumulation for ASNS-guided selection. EBV-associated T/NK-cell lymphoproliferative diseases may represent an emerging extension: in addition to a report describing successful bridging to allogeneic HSCT using ASNase monotherapy in acute fulminant chronic active EBV infection with markedly low ASNS expression at transcript and protein levels [56], Ichikawa et al. reported long-term disease control after allogeneic hematopoietic stem cell transplantation for nodal Epstein–Barr virus-positive T/NK-cell lymphoma in a case treated with L-asparaginase-containing initial chemotherapy, further supporting the relevance of ASNase-based strategies within EBV-driven T/NK proliferations, albeit in a broader transplant-oriented therapeutic context [57].

## 8. Pragmatic Clinical Use Model: A Minimal Decision Algorithm and Stopping Rules

A pragmatic clinical use model treats ASNS as an upfront screen (protein-first by IHC in ENKTL; intracellular flow on AML blasts where feasible), followed by disease-specific context filters that operationalize the “necessary-like but not sufficient” behavior of ASNS-low. In ENKTL, continuation should be governed by standardized response assessment (CR/PR) and durability metrics using Lugano criteria [58]. In AML, response categories should follow ELN-aligned definitions with explicit separation of CR from CRi, and early durability trajectory (DoR/EFS) should be prioritized over transient cytoreduction [59].

Feasibility constraints are central: the clinical utility of ASNS testing collapses if ASNase exposure cannot be maintained due to toxicity. Adult toxicity management frameworks emphasize hepatotoxicity, pancreatitis, hypertriglyceridemia, thrombosis, and hypersensitivity as key deliverability constraints [60]. ISTH guidance provides direction for prevention and management of asparaginase-related venous thromboembolism in adults [61], and large cohorts inform the risk and re-exposure considerations for asparaginase-associated pancreatitis [62]. Lack of objective response or early progression by standardized criteria, or inability to maintain exposure due to toxicity, should trigger switching to alternative regimens rather than prolonging non-durable amino-acid depletion.

## 9. Trial-Ready Companion Reagent: Validation Roadmap and Regulatory/QA Alignment

To make ASNS testing trial-ready, the near-term goal is a locked, auditable “ASNS-low” call with prespecified cutoffs, QC, and assay-change control. Evidence should be generated in staged designs: (i) biomarker-enriched phase II for signal finding, (ii) biomarker-stratified randomization to test treatment-biomarker interaction, and (iii) adaptive enrichment with prespecified rules to refine eligibility and thresholds [63,64]. Endpoints should cover analytical validity (reproducibility/robustness), clinical validity (response depth plus durability), and clinical utility (decision impact and net benefit) consistent with CDx expectations [42,43,44].

ENKTL is the anchor indication for prospective biomarker refinement because ASNase-containing regimens already have established activity [48,49,50]. AML is best pursued as a context-restricted extension in combination settings (e.g., venetoclax plus pegcrisantaspase) building on the translational signal reported by Emadi et al. [15] with prospective capture of escape mechanisms and microenvironmental confounders [8,15]. Routine deployment requires CAP-grade IHC validation and revalidation procedures [40,41], CLSI EP12-style evaluation of binary outputs [39], and medical-laboratory quality systems aligned to ISO 15189 [47], with forward planning for EU IVDR and EMA consultation processes when CDx claims are intended [45,46].

### Proposed Prospective Clinical Study Plan for ASNS-Guided Venetoclax–Asparaginase Therapy in AML

We propose a prospective biomarker-anchored phase Ib/II trial evaluating venetoclax–asparaginase combination therapy in biologically enriched AML populations. Eligible patients will include relapsed/refractory AML with complex karyotype, chromosome 7 abnormalities, TP53 mutation, or prospectively defined ASNS-low status by intracellular flow cytometry and/or validated immunohistochemistry.

**Figure 4:**
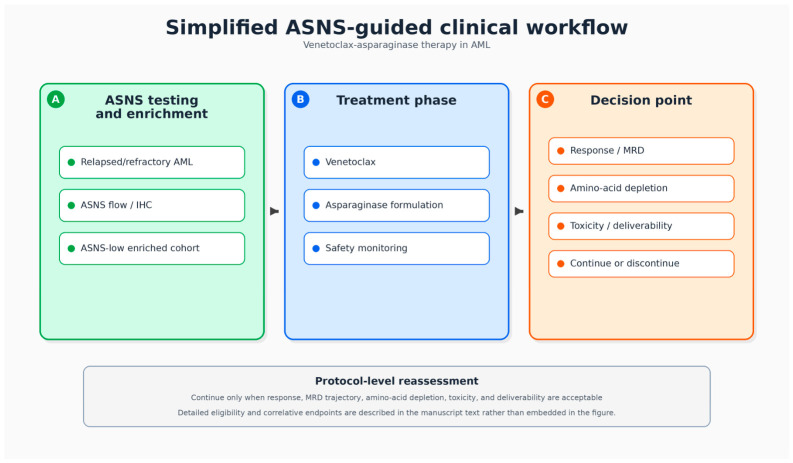
Simplified ASNS-guided clinical workflow for venetoclax–asparaginase therapy in AML. The figure summarizes a simplified three-step clinical development model for ASNS-guided venetoclax–asparaginase therapy in AML. (**A**) Patients with relapsed or refractory AML undergo ASNS assessment by intracellular flow cytometry and/or immunohistochemistry to identify an ASNS-low enriched cohort. (**B**) Eligible patients receive venetoclax plus an asparaginase formulation with protocol-defined safety monitoring. (**C**) Treatment continuation is determined by integrated assessment of early response, measurable residual disease, amino-acid depletion, toxicity, and treatment deliverability. Abbreviations: AML, acute myeloid leukemia; ASNS, asparagine synthetase; MRD, measurable residual disease.

A pragmatic next step is a multicenter, biomarker-anchored phase Ib/II study of venetoclax plus asparaginase in relapsed/refractory AML enriched for ASNS-low disease and other biologically plausible vulnerability states, including complex karyotype, TP53-mutated disease, and chromosome 7/7q-abnormal AML, building on the translational signal reported by Emadi et al. [15]. Primary objectives should be feasibility of repeated amino-acid depletion support and preliminary efficacy in the biomarker-enriched cohort, using composite remission rate with separate reporting of complete remission and complete remission with incomplete hematologic recovery [59]. Part A should establish a deliverable schedule with predefined monitoring for hepatotoxicity, pancreatitis, hypertriglyceridemia, thrombosis, bleeding risk, and hypersensitivity, whereas Part B should evaluate duration of response, event-free survival, overall survival, measurable residual disease conversion, and the proportion of patients maintaining protocol-specified depletion exposure [60,61,62]. Mandatory correlative studies should quantify ASNS by intracellular flow cytometry with reflex immunohistochemistry/proteomics, document plasma asparagine and glutamine depletion, and test whether benefit tracks more closely with static ASNS-low, low inducibility, or translational stress markers such as 4EBP1/eIF4E engagement and MCL-1 suppression [15] (Figure 4). A Simon two-stage or adaptive-enrichment design would permit early stopping for futility while prospectively refining the biomarker threshold [63,64].

## 10. Conclusions

A growing body of translational evidence suggests that asparagine synthetase (ASNS) expression reflects a dynamic metabolic state that shapes therapeutic vulnerability across hematologic malignancies. In this framework, reduced ASNS expression does not constitute a deterministic predictor of response but rather marks a lineage- and context-dependent condition of constrained metabolic adaptability. Such vulnerability appears to emerge from the interplay of differentiation-state-specific nutrient demands, adaptive stress-response signaling, and microenvironmental buffering of amino-acid availability.

Advances in protein-anchored diagnostics and rational combination design will pave the way for translating these metabolic dependencies into durable therapeutic successes. By enabling more precise mapping of metabolic phenotypes, these approaches may support rational integration of amino-acid depletion strategies with targeted therapies, including modulation of apoptotic pathways in myeloid disease and optimized multi-agent regimens in NK-lineage malignancies.

More broadly, the emerging ASNS paradigm highlights a shift from pathway-centric models of cancer metabolism toward a state-centric therapeutic logic, in which treatment vulnerability arises from limits in adaptive metabolic capacity rather than isolated enzymatic deficiency. Prospective, biomarker-anchored clinical investigation integrating multi-omic and pharmacodynamic endpoints will be required to determine whether such metabolically informed strategies can achieve durable clinical benefit. If validated, this conceptual framework may help bridge historically distinct therapeutic domains within hematologic oncology and inform the development of precision metabolic interventions across diverse malignancies.

## Figures and Tables

**Table 1:** AML subtype-specific metabolic context and potential vulnerability to asparagine depletion. Summary of AML subtypes and genetic contexts in which reduced ASNS expression or related metabolic features may increase susceptibility to L-asparaginase-mediated asparagine depletion. Evidence levels reflect currently available experimental, proteomic, or clinical observations; as classified based on availability of retrospective clinical cohorts vs. strictly in vitro perturbation data.

AML Subtype/Genetic Context	ASNS Expression Tendency	Biological Rationale for ASNase Sensitivity	Evidence Level
**M0/minimally differentiated AML**	Low–moderate	Immature metabolic phenotype with limited amino acid biosynthesis capacity	Emerging
**M1 AML**	Low	Early myeloid lineage state may rely more on extracellular amino acids	Emerging
**M5/monocytic AML**	Low	Monocytic leukemias exhibit altered amino-acid metabolism and stress-response signaling	Emerging
**Core-binding factor AML (inv(16))**	Reduced in subsets	Proteomic analyses suggest ASNS-low metabolic phenotype in part of this group	Emerging
**−7/del(7q) AML**	Low (ASNS haploinsufficiency)	Chromosome 7 deletions include the ASNS locus (7q21), increasing vulnerability to asparagine depletion	Preclinical/mechanistic
**RUNX1-mutated AML**	Variable but often reduced	RUNX1 dysregulation may influence metabolic gene networks including amino-acid metabolism	Hypothesis-generating
**Other AML subtypes**	Variable	Metabolic heterogeneity; ASNS expression may act as context-dependent biomarker	Hypothesis-generating

**Table 2:** Head-to-head comparison of methods to operationalize “ASNS-low” (analytical validity, deployability, and intended-use fit).

Assay Platform	Specimen	Output	Key Strengths	Main Failure Modes/ Confounders	Best-Fit Role (Near-Term)
**IHC/ICC (ASNS protein)**	FFPE tissue/cytology	Binary or semi-quantitative score	CDx-compatible; spatial context; low barrier	Fixation/processing, clone/retrieval dependence, scoring variability	Primary screen in ENKTL; outcome-anchored thresholding; CAP-grade validation
**Intracellular flow cytometry (ASNS protein)**	Fresh blood/marrow	ΔMFI/ratio on gated blasts	Single cell; rapid TAT; internal comparators	Permeabilization sensitivity; gating drift; lot effects	Primary screen in AML (trial/salvage); SOP lock + bridging controls
**Proteomics (RPPA/targeted MS)**	Frozen/FFPE; research cohorts	Relative or absolute abundance	Quantitative; scalable cohorts; multiplex context	Pre-analytic handling; digestion/LC variability; cost/TAT	Clinical validity support; threshold calibration; reference lab development
**Enzyme activity assay**	Fresh lysate	pmol Asn/min/mg (or proxy)	Mechanism-proximal reference	Specimen demands; low throughput; standardization burden	Reference standard for calibration and dispute resolution
**mRNA (qPCR/RNA-seq)**	Bulk tumor RNA	Expression level	High analytic precision; easy standardization	Tumor purity; mRNA-protein discordance; state dependence	Reflex/adjunct calibrated to protein or functional endpoints
**Promoter methylation (targeted)**	DNA from tumor cells	Methylation state	Stable state marker in defined contexts	Locus/lineage dependence; not universal	Adjunct in T-ALL-like epigenetic ASNS-off settings; harmonization aid

**Table 3:** Lineage- and cytogenetics-dependent patterns of ASNS expression and associated metabolic implications in acute myeloid leukemia.

AML Subset	ASNS Expression Pattern	Metabolic Implication	Therapeutic Implication	Evidence Source
**FAB M0**	Low	Immature metabolic program; reduced intrinsic asparagine biosynthesis	Potential sensitivity to ASNase-based depletion strategies	GEO transcriptome analysis
**FAB M1**	Low	Early myeloid differentiation state; amino-acid reliance	Candidate subset for metabolic targeting	GEO transcriptome analysis
**FAB M5 (monocytic)**	Low	Monocytic lineage metabolic plasticity; glutamine–asparagine coupling	Possible ASNase sensitivity; requires context-specific validation	GEO + translational literature
**FAB M4 (myelomonocytic)**	Moderate-high	Partial metabolic autonomy; enhanced amino-acid buffering	Potential relative resistance to ASNase monotherapy	GEO transcriptome analysis
**FAB M6 (erythroid)**	High	Differentiation-linked metabolic autonomy; increased biosynthetic capacity	Lower likelihood of ASNase sensitivity	GEO transcriptome analysis
**FAB M7 (megakaryoblastic)**	Intermediate	Lineage-specific metabolic heterogeneity	Variable therapeutic vulnerability	GEO transcriptome analysis
**inv(16) AML**	Markedly low	Core-binding factor AML with reduced ASNS biosynthetic capacity	Biologically plausible ASNase-sensitive subset	GEO + mechanistic models
**−7/del(7q) AML**	Very low	Gene-dosage-linked ASNS insufficiency; extracellular asparagine dependency	Strong candidate for ASNase-based metabolic therapy	GEO + translational case evidence
**Complex-karyotype AML**	Variable (often high stress-inducible)	ISR-driven adaptive ASNS induction	Combination of metabolic strategies required	Narayanan RPPA study [31]
**High-ASNS AML (proteomic)**	High	Shorter survival; adaptive metabolic stress buffering	Venetoclax response context-dependent	Narayanan [31]
**VEN-sensitive metabolic AML**	Context-dependent	Translational control & amino-acid signaling interplay	VEN + ASNase combination rationale	Liu [55]

## Data Availability

No new data were created or analyzed in this study. Data sharing is not applicable to this article.

## Abbreviations

ABC, antibody binding capacity; ALL, acute lymphoblastic leukemia; AML, acute myeloid leukemia; ASNase, L-asparaginase; ASNS, asparagine synthetase; ATF3, activating transcription factor 3; ATF4, activating transcription factor 4; ATF6, activating transcription factor 6; AUC, area under the curve; BCL-2, B-cell lymphoma 2; BM, bone marrow; CAP, College of American Pathologists; CDx, companion diagnostic; CLSI, Clinical and Laboratory Standards Institute; CR, complete remission; CRi, complete remission with incomplete hematologic recovery; CPTAC, Clinical Proteomic Tumor Analysis Consortium; ELN, European LeukemiaNet; EMA, European Medicines Agency; ENKTL, extranodal natural killer (NK)/T-cell lymphoma, nasal type; EFS, event-free survival; FAB, French–American–British; FFPE, formalin-fixed paraffin-embedded; GEO, Gene Expression Omnibus; HSCT, hematopoietic stem cell transplantation; ICC, immunocytochemistry; IC50, half-maximal inhibitory concentration; IHC, immunohistochemistry; ISR, integrated stress response; IVDR, In Vitro Diagnostic Regulation; LC–MS/MS, liquid chromatography–tandem mass spectrometry; LD70asp, 70% lethal dose of L-asparaginase; MCL-1, myeloid cell leukemia 1; MFI, mean fluorescence intensity; MIFlowCyt, Minimum Information about a Flow Cytometry Experiment; MNKPL, myeloid/NK-cell precursor acute leukemia; MS, mass spectrometry; NK, natural killer; PB, peripheral blood; PR, partial remission; QC, quality control; RPPA, reverse-phase protein array; TP53, tumor protein p53; UPR, unfolded protein response.

## References

[1] Su N., Pan Y.X., Zhou M., Harvey R.C., Hunger S.P., Kilberg M.S. (2008). Correlation between asparaginase sensitivity and asparagine synthetase protein content, but not mRNA, in acute lymphoblastic leukemia cell lines. Pediatr. Blood Cancer.

[2] Stams W.A.G., den Boer M.L., Beverloo H.B., Meijerink J.P.P., Stigter R.L., van Wering E.R., Janka-Schaub G.E., Slater R., Pieters R. (2003). Sensitivity to L-asparaginase is not associated with expression levels of asparagine synthetase in t(12; 21)^+^ pediatric ALL. Blood.

[3] Stams W.A.G., den Boer M.L., Holleman A., Appel I.M., Beverloo H.B., van Wering E.R., Janka-Schaub G.E., Evans W.E., Pieters R. (2005). Asparagine synthetase expression is linked with L-asparaginase resistance in TEL-AML1-negative but not TEL-AML1-positive pediatric acute lymphoblastic leukemia. Blood.

[4] Hermanova I., Zaliova M., Trka J., Starkova J. (2012). Low expression of asparagine synthetase in lymphoid blasts precludes its role in sensitivity to L-asparaginase. Exp. Hematol..

[5] Okada S., Hongo T., Yamada S., Watanabe C., Fujii Y., Ohzeki T., Horikoshi Y., Ito T., Yazaki M., Komada Y. (2003). In vitro efficacy of l-asparaginase in childhood acute myeloid leukaemia. Br. J. Haematol..

[6] Bertuccio S.N., Serravalle S., Astolfi A., Lonetti A., Indio V., Leszl A., Pession A., Melchionda F. (2017). Identification of a cytogenetic and molecular subgroup of acute myeloid leukemias showing sensitivity to L-asparaginase. Oncotarget.

[7] Noguchi K., Ikawa Y., Takenaka M., Sakai Y., Fujiki T., Kuroda R., Maeba H., Goto H., Kitoh T., Wada T. (2023). L-asparaginase as an efficient salvage therapy for refractory acute myeloid leukemia with chromosome 7 abnormalities: A case series. Int. J. Hematol..

[8] Michelozzi I.M., Granata V., De Ponti G., Alberti G., Tomasoni C., Antolini L., Gambacorti-Passerini C., Gentner B., Dazzi F., Biondi A. (2019). Acute myeloid leukaemia niche regulates response to L-asparaginase. Br. J. Haematol..

[9] Iwamoto S., Mihara K., Downing J.R., Pui C.H., Campana D. (2007). Mesenchymal cells regulate the response of acute lymphoblastic leukemia cells to asparaginase. J. Clin. Investig..

[10] Ehsanipour E.A., Sheng X., Behan J.W., Wang X., Butturini A., Avramis V.I., Mittelman S.D. (2013). Adipocytes cause leukemia cell resistance to L-asparaginase via release of glutamine. Cancer Res..

[11] Chen T., Zhang J., Zeng H., Zhang Y., Zhang Y., Zhou X. (2020). Antiproliferative effects of L-asparaginase in acute myeloid leukemia. Exp. Ther. Med..

[12] He Y., Li B., Zhang H., Luo C., Shen S., Tang J., Chen J. (2014). L-asparaginase induces apoptosis in AML U937 cells via an AIF-mediated mechanism. Front. Biosci..

[13] Chen H., Pan Y.X., Dudenhausen E.E., Kilberg M.S. (2004). Amino acid deprivation induces the transcription rate of the human asparagine synthetase gene through a timed program of expression and promoter binding of nutrient-responsive basic region/leucine zipper transcription factors as well as localized histone acetylation. J. Biol. Chem..

[14] Gjymishka A., Su N., Kilberg M.S. (2009). Transcriptional induction of the human asparagine synthetase gene during the unfolded protein response does not require the ATF6 and IRE1/XBP1 arms of the pathway. Biochem. J..

[15] Emadi A., Kapadia B., Bollino D., Bhandary B., Baer M.R., Niyongere S., Strovel E.T., Kaizer H., Chang E., Choi E.Y. (2021). Venetoclax and pegcrisantaspase for complex karyotype acute myeloid leukemia. Leukemia.

[16] Chan W.K., Horvath T.D., Tan L., Link T., Harutyunyan K.G., Pontikos M.A., Anishkin A., Du D., Martin L.A., Yin E. (2019). Glutaminase activity of L-asparaginase contributes to durable preclinical activity against acute lymphoblastic leukemia. Mol. Cancer Ther..

[17] Krall A.S., Xu S., Graeber T.G., Braas D., Christofk H.R. (2016). Asparagine promotes cancer cell proliferation through use as an amino acid exchange factor. Nat. Commun..

[18] Chang M.C., Staklinski S.J., Merritt M.E., Kilberg M.S. (2023). A method for measurement of human asparagine synthetase activity and application to ASNS protein variants associated with ASNS deficiency. Biol. Methods Protoc..

[19] Schwartz R.S. (1969). Immunosuppression by L-asparaginase. Nature.

[20] Broome J.D. (1963). Evidence that the L-asparaginase of guinea pig serum is responsible for its antilymphoma effects: II. Lymphoma 6C3HED cells cultured in a medium devoid of L-asparagine lose their susceptibility to the effects of guinea pig serum in vivo. J. Exp. Med..

[21] Takahashi H., Koh K., Kato M., Kishimoto H., Oguma E., Hanada R. (2012). Acute myeloid leukemia with mediastinal myeloid sarcoma refractory to AML therapy but responsive to L-asparaginase. Int. J. Hematol..

[22] Kitoh T., Sawada M., Horikoshi Y., Miyake M., Kanai R., Ogawa A., Asami K., Kamitamari A., Tsumsawa M., Fujimoto T. (2000). Clinical application of L-asparaginase to intractable acute non-lymphocytic leukemia. Med. Pediatr. Oncol..

[23] Morimoto M., Kondoh K., Keino D., Ohyama R., Ban S., Kinoshita A., Kitoh T. (2010). A child with myeloid/natural killer cell precursor acute leukemia treated successfully with AML-oriented chemotherapy incorporating L-asparaginase. Leuk. Res..

[24] Horikoshi A., Takei K., Iriyama N., Uenogawa K., Ishizuka H., Shiraiwa H., Hosokawa Y., Sawada S., Kitoh T. (2009). Effect of L-asparaginase combined with vincristine and prednisolone on acute myeloblastic leukemia (M0) associated with non-Hodgkin lymphoma. Acta Haematol..

[25] Tezuka K., Nakayama H., Honda K., Suzumiya J., Oshima K., Kitoh T., Ishii E. (2002). Treatment of a child with myeloid/NK cell precursor acute leukemia with L-asparaginase and unrelated cord blood transplantation. Int. J. Hematol..

[26] Hatta S., Kitoh T., Irino T., Umemura S., Mukai K., Osaka M., Suzuki T. (2005). Establishment of flow cytometrical detection of asparagine synthetase protein in leukemia cells. Cytom. Res..

[27] Kusano-Arai O., Iwanari H., Mochizuki Y., Nakata H., Kodama T., Kitoh T., Hamakubo T. (2012). Evaluation of the asparagine synthetase level in leukemia cells by monoclonal antibodies. Hybridoma.

[28] Kitoh T., Siqiang K.S.G., Kato H., Miyata K., Shimomura Y., Hori T., Sakaguchi K., Horikoshi Y., Ohki K., Koh K. (2014). Flow cytometric detection of asparagine synthetase protein in leukemia cells: Indication for L-asparaginase therapy. Blood.

[29] Lee J.A., Spidlen J., Boyce K., Cai J., Crosbie N., Dalphin M., Furlong J., Gasparetto M., Goldberg M., Goralczyk E.M. (2008). MIFlowCyt: The minimum information about a flow cytometry experiment. Cytom. A.

[30] Kalina T., Flores-Montero J., van der Velden V.H.J., Martin-Ayuso M., Böttcher S., Ritgen M., Almeida J., Lhermitte L., Asnafi V., Mendonça A. (2012). EuroFlow standardization of flow cytometer instrument settings and immunophenotyping protocols. Leukemia.

[31] Narayanan N., Marvin-Peek J., Abouelnaaj M.K., Majid D., Wang B., Brown B.D., Qiu Y., Kornblau S.M., Abbas H.A. (2024). Reverse Phase Proteomic Array Profiling of Asparagine Synthetase Expression in Newly Diagnosed Acute Myeloid Leukemia. J. Proteome Res..

[32] Abbatiello S.E., Pan Y.X., Zhou M., Wayne A.S., Veenstra T.D., Hunger S.P., Kilberg M.S., Eyler J.R., Richards N.G., Conrads T.P. (2008). Mass spectrometric quantification of asparagine synthetase in circulating leukemia cells from acute lymphoblastic leukemia patients. J. Proteom..

[33] Gerber S.A., Rush J., Stemman O., Kirschner M.W., Gygi S.P. (2003). Absolute quantification of proteins and phosphoproteins from cell lysates by tandem MS. Proc. Natl. Acad. Sci. USA.

[34] Xuan Y., Bateman N.W., Gallien S., Goetze S., Zhou Y., Navarro P., Hu M., Parikh N., Hood B.L., Conrads K.A. (2020). Standardization and harmonization of distributed multi-center proteotype analysis supporting precision medicine studies. Nat. Commun..

[35] Whiteaker J.R., Halusa G.N., Hoofnagle A.N., Sharma V., MacLean B., Yan P., Wrobel J.A., Kennedy J., Mani D.R., Zimmerman L.J. (2016). Using the CPTAC Assay Portal to Identify and Implement Highly Characterized Targeted Proteomics Assays. Methods Mol. Biol..

[36] Liu W.J., Wang H., Peng X.W., Wang W.D., Liu N.W., Wang Y., Lu Y. (2018). Asparagine synthetase expression is associated with the sensitivity to asparaginase in extranodal natural killer/T-cell lymphoma in vivo and in vitro. OncoTargets Ther..

[37] Touzart A., Lengliné E., Latiri M., Belhocine M., Smith C., Thomas X., Spicuglia S., Puthier D., Pflumio F., Leguay T. (2019). Epigenetic silencing affects L-asparaginase sensitivity and predicts outcome in T-ALL. Clin. Cancer Res..

[38] Zhou R., Liang T., Li T., Huang J., Chen C. (2023). Possible mechanism of metabolic and drug resistance with L-asparaginase therapy in childhood leukaemia. Front. Oncol..

[39] Clinical and Laboratory Standards Institute (CLSI) (2023). Evaluation of Qualitative, Binary Output Examination Performance.

[40] Fitzgibbons P.L., Bradley L.A., Fatheree L.A., Alsabeh R., Fulton R.S., Goldsmith J.D., Haas T.S., Karabakhtsian R.G., Loykasek P.A., Marolt M.J. (2014). Principles of analytic validation of immunohistochemical assays: Guideline from the College of American Pathologists Pathology and Laboratory Quality Center. Arch. Pathol. Lab. Med..

[41] Goldsmith J.D., Troxell M.L., Roy-Chowdhuri S., Colasacco C.F., Edgerton M.E., Fitzgibbons P.L., Fulton R., Haas T., Kandalaft P.L., Kalicanin T. (2024). Principles of Analytic Validation of Immunohistochemical Assays: Guideline Update. Arch. Pathol. Lab. Med..

[42] U.S. Food and Drug Administration (2014). In Vitro Companion Diagnostic Devices: Guidance for Industry and FDA Staff.

[43] U.S. Food and Drug Administration (2023). Companion Diagnostics.

[44] U.S. Food and Drug Administration (2026). List of Cleared or Approved Companion Diagnostic Devices.

[45] The European Parliament, The Council of the European Union (2017). Regulation (EU) 2017/746 of the European Parliament and of the Council of 5 April 2017 on in vitro diagnostic medical devices (IVDR). Off. J. Eur. Union.

[46] European Medicines Agency (EMA) (2022). Guidance on the Procedural Aspects for the Consultation to the EMA by a Notified Body on Companion Diagnostics.

[47] (2022). Medical Laboratories—Requirements for Quality and Competence.

[48] Yamaguchi M., Kwong Y.L., Kim W.S., Maeda Y., Hashimoto C., Suh C., Izutsu K., Ishida F., Isobe Y., Sueoka E. (2011). Phase II study of SMILE chemotherapy for newly diagnosed stage IV, relapsed, or refractory extranodal NK/T-cell lymphoma, nasal type: The NK-Cell Tumor Study Group study. J. Clin. Oncol..

[49] Jaccard A., Gachard N., Marin B., Rogez S., Audrain M., Suarez F., Tilly H., Morschhauser F., Thieblemont C., Ysebaert L. (2011). Efficacy of L-asparaginase with methotrexate and dexamethasone (AspaMetDex regimen) in patients with refractory or relapsing extranodal NK/T-cell lymphoma: A phase II study. Blood.

[50] Wang J.H., Wang H., Wang Y.-J., Xia Z.-J., Huang H.-Q., Jiang W.-Q., Lu Y. (2016). Analysis of the efficacy and safety of a combined gemcitabine, oxaliplatin and pegaspargase regimen for NK/T-cell lymphoma. Oncotarget.

[51] Ando M., Sugimoto K., Kitoh T., Sasaki M., Mukai K., Ando J., Egashira M., Schuster S.M., Oshimi K. (2005). Selective apoptosis of natural killer-cell tumours by L-asparaginase. Br. J. Haematol..

[52] Matsumoto Y., Nomura K., Kanda-Akano Y., Fujita Y., Nakao M., Ueda K., Horiike S., Yokota S., Kusuzaki K., Kitoh T. (2003). Successful treatment with Erwinia L-asparaginase for recurrent natural killer/T cell lymphoma. Leuk. Lymphoma.

[53] Sakamoto E., Yamane T., Nakane T., Takeoka Y., Hirose A., Hagihara K., Nakamae H., Ohta K., Hirayama M., Ikura Y. (2005). Temporary effective treatment with L-asparaginase for a patient with refractory nasal NK/T-cell lymphoma. Gan To Kagaku Ryoho.

[54] Xiao Y., Hu B., Guo Y., Zhang D., Zhao Y., Chen Y., Li N., Yu L. (2023). Targeting glutamine metabolism as an attractive therapeutic strategy for acute myeloid leukemia. Curr. Treat. Options Oncol..

[55] Liu Y., Bollino D.R., Bah O.M., Strovel E.T., Le T.V., Zarrabi J., Philip S., Lapidus R.G., Baer M.R., Niyongere S. (2025). A phase 1 study of the amino acid modulator pegcrisantaspase and venetoclax for relapsed or refractory acute myeloid leukemia. Blood.

[56] Furukawa Y., Ando J., Ishii M., Kinoshita S., Goto A., Tachibana K., Azusawa Y., Kato T., Izumi N., Hosoya E. (2024). L-asparaginase monotherapy as an encouraging approach towards acute fulminant chronic active Epstein-Barr virus infection. Br. J. Haematol..

[57] Ichikawa S., Abe H., Morota N., Kawajiri A., Nakagawa R., Inokura K., Hatta S., Katsuoka Y., Onodera K., Fukuhara N. (2025). Successful Allogeneic Hematopoietic Stem Cell Transplantation for Nodal Epstein-Barr Virus-positive T/NK-cell Lymphoma. Intern. Med..

[58] Cheson B.D., Fisher R.I., Barrington S.F., Cavalli F., Schwartz L.H., Zucca E., Lister T.A. (2014). Recommendations for initial evaluation, staging, and response assessment of Hodgkin and non-Hodgkin lymphoma: The Lugano classification. J. Clin. Oncol..

[59] Döhner H., Wei A.H., Appelbaum F.R., Craddock C., DiNardo C.D., Dombret H., Ebert B.L., Fenaux P., Godley L.A., Hasserjian R.P. (2022). Diagnosis and management of AML in adults: 2022 recommendations from an international expert panel on behalf of the ELN. Blood.

[60] Burke P.W., Hoelzer D., Park J.H., Schmiegelow K., Douer D. (2020). Managing toxicities with asparaginase-based therapies in adult ALL: Summary of an ESMO Open-Cancer Horizons roundtable discussion. ESMO Open.

[61] Zwicker J.I., Wang T., DeAngelo D.J., Lauw M.N., Connors J.M., Falanga A., McMasters M., Carrier M. (2020). The prevention and management of asparaginase-related venous thromboembolism in adults: Guidance from the SSC on Hemostasis and Malignancy of the ISTH. J. Thromb. Haemost..

[62] Rank C.U., Wolthers B.O., Grell K., Albertsen B.K., Frandsen T.L., Overgaard U.M., Toft N., Nielsen O.J., Wehner P.S., Harila-Saari A. (2020). Asparaginase-associated pancreatitis in acute lymphoblastic leukemia: Results from the NOPHO ALL2008 treatment of patients 1-45 years of age. J. Clin. Oncol..

[63] U.S. Food and Drug Administration (2019). Enrichment Strategies for Clinical Trials to Support Approval of Human Drugs and Biological Products: Guidance for Industry.

[64] U.S. Food and Drug Administration (2019). Adaptive Design Clinical Trials for Drugs and Biological Products: Guidance for Industry.

